# 

**Published:** 1844-01-01

**Authors:** 


### MEDICO-CHIRURGICAL

### REVIEW,

AND

### JOURNAL

OF

### PRACTICAL MEDICINE.

*(NEW SERIES.)*

VOLUME FORTY,

[1st of OCTOBER, 1843, to 31st of MARCH,]

1844.

VOL. XX. of DECENNIAL SERIES.

nothing to index

EDITED

By JAMES JOHNSON, M.D.

PHYSICIAN EXTRAORDINARY TO THE LATE KING,

AND OTHERS.

Surgeon General's Office
LIBRARY
57480.
Washington, D.C.

LONDON:
PUBLISHED BY S. HIGHLEY, 32, FLEET STREET,
*Re-printed in New York, by Mr. Wood.*
1844.

WI
ME 75 T

PRINTED BY F. HAYDEN,

Little College Street, Westminster.

### INDEX.

A.

Abscess in Schneiderian membrane.. 564
Absence of the rectum ............ 541
Acardiac fœtuses, circulat. of blood in, 517
Acceleration of puberty in the female, 463
Accessory nerve, functions of the.... 504
Aconitine in facial neuralgia........ 548
Action of medicines .............. 181
Addison on pneumonia............ 241
Addison on pneumonia............ 464
Adventitious structures, Hodgkin on, 378
Age, Beau on the diseases of........ 491
Age for females to marry .......... 516
Air, Detmold on highly condensed .. 276
Alcohol and ether, action of........ 181
Aldridge on chyle ................ 522
Aldridge on urinary diseases........ 528
Aliments ........................ 410
Amaurosis ...................... 349
Amenorrhœa, on the cause of ...... 208
Ammonia in scarlatina, Rieken on .. 454
Amniotic fluid, asphyxia produced by, 514
Anæmia ........................ 303
Analogy between monstrosities ..... 507
Anatomy, formative and structural .. 1
Anatomy of the Chinese .......... 505
Anatomy, philosophical..... ...... 507
Andral on pathological hæmatology.. 469
Anecdote, medical ............... 239
Aneurysm of the umbilical cord .... 538
Angina, epizootic gangrenous ...... 235
Animal physiology, Carpenter's .... 1
Animal kingdom, development of the, 11
Animal heat .................... 401
Ankylosis, diagnosis of true and false, 261
Anodyne pommade ............... 515
Anterior chamber of eye .......... 556
Antimony in injuries and ophthalmia, 512
Anti-scrofulous syrup ............ 516
Apothecaries' diploma in Paris...... 575
Arch-tourniquet.................. 263
Arsenic, hydrated peroxide of iron in poisoning by .................. 280
Arteries, fatty degeneration of the .. 39
Artificial pupil, operation for ....... 348
Asphyxia........................ 229
Asphyxia from amniotic fluid ...... 514
Atlee on extirpation of ovarian cysts, 258
Auscultation, obstetrical, Arsdale's .. 269
Auscultation, clearness of sounds of.. 537
Axilla, medication by the .......... 237

B.

Bagneres de Bigorre ............... 54
Ballingall on naval & military schools, 355
Barbadoes leg.................... 509
Barreges ..................... ... 57
Battley on yellow bark ............: 277
Beau on the diseases of old age...... 491
Beck on opium in infants .......... 545
Becquerel's semeiology of the urine.. 185
Bed-sores, nitrate of silver for ...... 267
Bell on an epidemic at Teheran...... 383
Bell on naval and military surgery .. 443
Bennett on parasitic veget. structures, 456
Bennett on inflam. of nervous centres, 459
Benzoin water .................... 578
Bethlem, Webster's statistics of .... 364
Bichat, statue to ........... ..... 239
Bichromate of potass, poisoning by .. 551
Bird on fatty urine................ 276
Bird's natural philosophy .......... 391
Bischoff on fecundation............ 206
Bisulphuret of carbon ............. 240
Blood, compression of, Robinson on, 38
Blood, alterations of the .......... 147
Blood and urine of chlorotic subjects, 179
Blood, determination of............ 307
Blood, microscopic entozoa in the.... 478
Blood, Donné on the............... 499
Blood, circulat. of, in acardiac fœtuses 517
Blood, oxides of protein in ........ 520
Blood in anterior chamber.......... 556
Bloxam's case of ulcer. of int. jugular, 42
Bone, Simon on diseases of ........ 87
Bones, morbid structures of........ 94
Bony texture, diseases of the ....... 92
Boudet on phthisis................ 217
Bouisson on dislocations .......... 410
Bowels of the horse, verm. tumors in, 236
Bowman's physiological anatomy.... 1
Breast, cancer of the .............. 122
Brodie on foreign bodies in bronchus, 372
Brodie on surgical practice ......... 564
Bronchial calculus, Tice's case of.... 34
Bronchocele, treatment of.......... 121
Buel on a peculiar form of paralysis.. 534
Burns, treatment of, by sod. carb. ... 556
Burton on the statistics of fever...... 531

C.

Cabanis, works of . .............. 494
Cæcum, protrusion of, on the left side, 262
Calculi in St. George's, Jones on the, 39
Calculi, new instrument for crushing, 576
Calomel in typhoid fever .......... 234
Caloric, Metcalfe on .............. 391
Caloric, the source of motion ....... 398
Caloric, its identity with electricity.. 399
Caloric, its influence on digestion.... 403
Caloric, its influence on nutrition.... 404
Caloric, production of sleep by...... 411
Caloric, its influence on spasmodic dis. 413
Caloric, its influence on inflammation, 414
Cancer, cell-formation of .......... 28
Cancer, Tuson on the treatment of.. 547
Cancerous diseases, frequency of.... 213
Cancrum oris, Dr. Hunt on ........ 44
Cannabis Indica, Clendinning on the, 376
Capbern, waters of................ 57

Carbon, bisulphuret of ............ 240
Caries of the os calcis.............. 263
Carotids, compres. of in hydrophobia, 265
Carpenter's animal physiology ...... 1
Cartilage, development of......... 27
Catamenia, influence of opium on the, 545
Cataract, Scott on.. ............ 190
Cataract knife, description of Scott's, 190
Catarrh, Watson on ............. 166
Cattle, Dumas on the fattening of.... 230
Cauterets, waters of .............. 58
Cayol on the vis medicatrix naturæ.. 218
Cell-formation, doctrine of ........ 1
Cells, Williams on the pathology of.. 246
Ceratum saponis, Houlton on ...... 279
Cerebral softening, nature of........ 459
Cerebral softening, colour of........ 461
Cerebral softening, connexion between hæmorrhage, and ............... 462
Chadwick's sanitary report ........ 415
Chancre, tanning a................ 548
Chapman on phlebitis ............. 259
Chaps, pommade for............. 515
Charter of Veterinary College ...... 579
Chemical analysis, Fresenius on .... 179
Chemical pathology ............... 480
Chemistry, Kane's elements of...... 185
Chervin, death of .............. 195
Chinese, anatomy of the .......... 505
Chlorotic subjects, blood and urine of, 179
Chloruretted lotions in variola...... 513
Churchill on dis. of uterine appendages 270
Chyle, composition of ............. 24
Chyle, Aldridge on................ 522
Civil government, Foltz on ........ 283
Classical illustrations of diseases .... 203
Clayton on hysterical affection of voice 42
Climate, influence of.............. 405
Climate of France, variations in the.. 497
Climate of Canada, Orton on the.... 546
Clinical medicine, Graves'.......... 163
Cochineal in hooping-cough........ 552
Colostrum, constitution of the ...... 503
Comparative anatomy, Owen on .... 1
Complicated menstruat. Detmold on, 536
Compression of the blood, Robinson on 38
Compression for urethral pains...... 514
Confectio ferri composita.......... 553
Congenital luxations, Gerdy on ..... 512
Congestion ...................... 305
Congestive pneumonia, Erichsen on, 36
Conjunctiva, strumous inflam. of the, 114
Cooper on lithotomy .............. 241
Copaiba lozenges ............... 515
Cornea, opacities of the........... 347
Corpora lutea, Raciborski on the.... 486
Critical age in women, Raciborski on, 211
Croton oil, inoculation of, for nævus, 512
Crowfoot's case of ulcer. of pul. artery 45
Cryptogamic plants in sputa of phthisis 457
Cryptogam. plants in skin of gold fish, 457
Curling on diseases of the testis ..... 313
Cutaneous diseases, memoranda on .. 237

D.

Dalrymple on ossific. of encysted tum. 372
Dartos, muscularity of the ......... 314
Dauverger on varus mentagra ...... 265
Decomposition of phosphatic calculi.. 191
Delirium, musk in ................ 511
Detmold on complicated menstruation 536
Detmold on highly-condensed air ... 276
Dickson, Dr. on trees & shrubs for cem. 579
Digestion, influence of caloric on ... 403
Dislocation of the 4th thoracic cartilage 440
Dislocation, sub-acromial, of humerus 441
Dislocation forwards of up. end of ulna 442
Dislocation directly backw. of femur, 442
Dog, rabies in the ................ 515
Doherty on impending dissolution .. 533
Donné on micros. examination of fluids 498
Dover's powder, poisoning by ...... 550
Dropsy, Watson on ............... 163
Dropsy, treatment of.............. 310
Dropsy of the uterus.............. 487
Dumas on the fattening of cattle .... 230
Duvergie on infanticide............ 227
Dying, different modes of .......... 149

E.

Ear, Toynbee on the pathology of the, 369
Eaux-bonnes and Eaux-chaudes .... 59
Eczema, Johnson on .............. 254
Egypt, medical school in .......... 496
Electricity in poisoning by laudanum, 544
Electro-puncture in hydrocele ...... 260
Elephantiasis, case of.............. 509
Elephantiasis, tubercular, Kinnis on, 530
Emengard's nosologie clinique ...... 496
Empyema of necessity, pulsating .... 540
Encysted hydrocele, Liston on ...... 361
Encysted tumors, Dalrymple on .... 372
Entozoa in the blood and stomach .. 478
Entozoa, intestinal, during digestion, 479
Epidemic mortality in England...... 61
Epidemic at Teheran, Bell on an .... 383
Epilepsy, simulation of ... ........ 84
Epistaxis, cure of ................ 268
Epithelium-cells of the intestines .... 236
Epizootic gangrenous angina ....... 235
Erectile tumor in the popliteal space, 43
Erichsen on congestive pneumonia .. 36
Eruptive fevers, Gregory on the .... 60
Excision of the uterus ............ 257
Exercise, influence of, on respiration, 409
Exophthalmia from hydatid in the orbit 478
Exostosis.................. ....... 91
Extirpation of the sup. maxilla, case of 249
Extirpation of ovarian cysts, Key on, 250
Extirpation of ovar. cysts, Walne on, 258
Extirpation of ovar. cysts, Atlee on.. 258
Extirpation of ovarian tumors ...... 557
Eye, Hall on diseases of the ........ 111
Eye, malignant diseases of the ...... 119
Eye, periodic affections of the ...... 228
Eyebrows and eyelashes, loss of ..... 573

F.

Face and head, tic douloureux of the 236
Face, contraction of the muscles of.. 562
Facial neuralgia, aconitine in........ 518
Fæculent discharge at the umbilicus, King on .................... 245
Fattening of cattle, Dumas on the .. 231
Fatty degeneration of arteries, Gulliver 39
Fatty urine, Dr. Bird on .......... 276
Feigned diseases, Gavin on ........ 69
Female, Mercier on gonorrhœa in the 451
Femur, dislocation of, on dorsum ilii 264
Fever, theory of .................. 413
Fever, Burton on the statistics of.... 531
Field on veterinary surgery ........ 125
Fife on caries of the os calcis ...... 263
Fissured palate, operation for........ 262
Fissures of the toes, pommade for .. 515
Fistula in ano.... ................ 120
Fluids, microscopic examinations of.. 499
Fœcundation, Bischoff on . ........ 206
Fœcundation in mammiferous animals 223
Foltz's medical statistics .......... 281
Foltz on civil government.......... 283
Foreign bodies in bronchus, Brodie on 372
Forensic medicine, Guy's .......... 183
Fracture of the base of the skull.... 508
France, on the use of ptisans in .... 481
France, variations in the climate of.. 497
Fresenius on chemical analysis ...... 179
Frogley on osteosarcoma of the thigh 44

G.

Galen on the hand ................ 453
Geography, medical .............. 235
Gerdy on congenital luxations ...... 512
German hydropathic journal........ 514
Glanders ........................ 128
Gonorrhœa in the female, Mercier on 451
Gout, Graves on.................. 171
Gouty ramollissement of spinal cord 173
Graves' clinical medicine .......... 143
Gregory on the eruptive fevers...... 60
Gun-shot wounds . .............. 437
Guthrie on the urinary organs ...... 183
Guy's forensic medicine............ 183
Guy's hospital reports ...... ..... 241

H.

Hæmatology, pathological, Andral on 469
Hæmorrhage, proximate cause of.... 237
Hæmorrhage, Watson on .......... 162
Hæmospasic treatment ............ 231
Halford, Sir Henry .............. 580
Hall on diseases of the eye . ...... 111
Hall on lateral curvature .......... 556
Halpin on puerperal convulsions .... 271
Hand, Galen on the .............. 453
Harris on puerperal convulsions .... 268
Health and disease, Reveillé-Parise on 197
Health, influence of manufactures on 462
Healthy and diseased bone, Simon on 87
Heart, Pigeaux on diseases of the.... 490
Heat and cold, effects of .......... 150
Hermaphroditism, Knox on . ...... 523
Hoarseness ...................... 175
Hodgkin on adventitious structures .. 378
Holland on mechanical disease of lungs 452
Hooping cough, cochineal in........ 552
Horse, hepatic disease in the........ 135
Horse, intestinal diseases in the .... 136
Horse, cerebral diseases in the ...... 139
Horse, urinary diseases in the ...... 141
Horse, verminous tumors in the .... 236
Horses, influenza among .......... 133
Hoskins on decomposition of phosphatic calculi .................. 191
Houlton on ceratum saponis........ 279
Houston on acardiac fœtuses........ 517
Humoralism, King on ............ 525
Hunt on cancrum oris ............ 44
Hydatid in orbit, exophthalmia from 478
Hydatids, transmission of,by contagion 473
Hydatids, false or spurious ........ 473
Hydatids, polycephalus ............ 473
Hydatids, ætiology of.............. 475
Hydrocele, electro-puncture in...... 260
Hydrocele, encysted, Liston on...... 361
Hydrocele, spermatozoa in the fluid of 362
Hydropathic treatment, death from .. 577
Hydrophobia, treatment of, by compression of the carotids.......... 265
Hyperæmia ...................... 304
Hypertrophy and atrophy of tissues.. 144
Hysterical affection of vocal apparatus 42

I & J.

Imprisonment, solitary, with labour.. 493
Indian medical service ........ 289, 581
Indian flesh-glove ................ 291
Infantile paralysis ................ 512
Infanticide, Duvergie on .......... 227
Infants, nervous affections in...... .. 533
Infants, Beck on opium in.......... 545
Infæcundity of females ............ 519
Inflammation, Watson on.......... 154
Inflammation, theory of............ 414
Inflammation of nervous centres, Bennett on....... ............... 459
Influenza among horses............ 133
Injections, warm, in stricture. ...... 261
Iron, hydrated peroxide of, in poisoning by arsenic .................. 280
Insanity, Webster on.............. 364
Insanity, proneness of women to.... 365
Insanity, suicidal ........ ...... 366
Insanity, pathological changes in .... 367
Interments in cities .............. 416
Interments, delay of .............. 423
Intestinal entozoa during digestion .. 479
Intestines, epithelium-cells of the.... 236
Intolerance of light, cause of........ 345
Invalids, hints to ................ 50
Invertebrate animals, comp. anat. of .. 1
Johns on puerperal convulsions...... 271
Johnson, on ulcers of the legs ...... 254
Johnson on eczema................ 254

Jones on the calculi in St. George's.. 39
Jones on the human ovum.......... 272
Jugular ulceration of the .......... 42
Jurisprudence, Taylor's medical . .. 432
K.
Kane's elements of chemistry ...... 184
Key on extirpation of ovarian cysts.. 250
Kheesah, the .................... 291
King on a feculent discharge at the umbilicus.................... 245
King on humoralism .............. 525
Kinnis on tubercular elephantiasis.... 530
Knox on hermaphroditism.......... 523
L.
Labouring population, condition of the 415
Lane on matico .................. 278
Lateral curvature of the spine . .... 221
Lateral curvature, Hall on.......... 556
Laudanum, electricity in poisoning by 544
Leeches, preservation of............ 552
Leeches good weather-guages....... 575
Legs, ulcers of the, Johnson on .... 254
Leprosy in Sardinia .............. 509
Lever on diseases of the uterus...... 98
Lever on puerperal convulsions .... 251
Liebig's theory of urinary secretion . 528
Liston's case of erectile tumor ...... 43
Liston on encysted hydrocele........ 361
Lithotomy, Cooper on ............ 241
Lithotrity, how to perform.......... 242
Lombard on typhus fever .......... 500
Luke's case of strangulated hernia reduced "en masse" .............. 45
Lungs, Holland on mechanical disease of 452
Luxations, congenital, Gerdy on .... 512
M.
Malaria ........................ 408
Mammiferous animals, fecundation in 223
Man, Jones on the ovum of ........ 272
Manufactures, Noble on............ 462
Marriage, remarks on.............. 226
Maschaliatria .................... 237
Matico, Lane on.................. 278
Medical memoranda .............. 229
Medical geography ................ 235
Medical anecdote.................. 239
Medical statistics, Foltz's .......... 281
Medical jurisprudence, Taylor's...... 432
Medical education in the United States 570
Medical schools in England & America 572
Medical remuner. in emigrant vessels, 574
Medication by the axilla .......... 237
Medicine, Williams' principles of.... 292
Medicines, on the action of ........ 181
Medico-chirurgical transactions ..31, 361
Memoranda on cutaneous diseases.... 237
Menstruation, Remak on .......... 484
Mental faculties, action of medicines on 489
Mercury and quina, new salt of...... 550
Metcalfe on caloric................ 391
Meteorology .................... 543
Metorrhagia, opiates in ............ 489
Metropolis, mortality in the........ 543
Microscopic examination of tartar.... 234
Microscopic examination of fluids .. 499
Milk, microscopic examination of.... 503
Minor surgery, Smith's............ 468
Molluscum ...................... 239
Monstrosities, analogy between...... 507
Morality versus physiology ........ 275
Mortality, quarterly table of........ 541
Mucus, microscopic examination of.. 501
Mulder on the blood .............. 520
Musk in delirium ................ 511
N.
Nævi, inoculation of croton oil in.... 513
Naphtha in phthisis .............. 267
Naval & military schools, Ballingall on 355
Naval and military surgery, Bell on.. 443
Natural philosophy, Dr. Bird's ...... 391
Neuralgia, inoculation of veratria in.. 510
Neuralgia, nux vomica in .......... 510
Nerves, properties of the .......... 298
Nervous and sanguiferous systems, reciprocal influence of the.......... 229
Nervous centres, Bennett on inflammation of the .................. 459
Nervous affections in infants........ 533
New truss ...................... 264
Nitrate of silver for bed sores ... .. 267
Nitrate of potass, on large doses of .. 549
Nosologie clinique, Emengard's...... 496
O.
Obstetrical auscultation, VanArsdale on 269
Ocular inflammation, remedies in.... 113
Oculist's vade-mecum, Walker's .... 341
Opacities of the cornea............ 347
Ophthalmia, irritable of females .... 116
Ophthalmia, purulent ............ 117
Ophthalmia, chronic, treatment of .. 342
Ophthalmia, nitrate of silver in .... 511
Ophthalmia, antimony in .......... 512
Ophthalmic surgery .............. 341
Opiates in metorrhagia ............ 489
Opium, influence of, on the catamenia 545
Opium in the infant subject ........ 545
Opposition coach.................. 285
Orthopædic controversy in Paris .... 201
Orton on the climate of Canada .... 546
Os calcis, Fife on caries of the...... 263
Osann, last illness of Dr. .......... 193
Ossification of encysted tumors, Dalrymple on the.................. 372
Osteitis ........................ 92
Osteophyte ...................... 91
Osteoporosis .................... 93
Osteosarcoma of thigh, Frogley's cases 44
Ovarian cysts, Key on extirpation of 250
Ovarian cysts, Walne on extirpation of 258
Ovarian cysts, Atlee on extirpation of 258
Ovarian tumors, extirpation of...... 557
Owen on comparative anatomy...... 1
Ovum of man & mammifera, Jones on 272
Oxydes of protein in the blood...... 520

P

Palate, fissure of, operation for...... 262
Paralysis from disease of cervical medulla ........................ 32
Paralysis in infancy .............. 512
Paralysis, peculiar form of, ......... 536
Parasitic vegetable structures ...... 456
Paris, orthopædic controversy in .... 201
Paris, apothecaries' diploma in....... 575
Pathogeny .................. .... 296
Pathology, application of cell-doctrine in 28
Pathology of cells, Williams on...... 246
Pau, Taylor on the climate of ...... 49
Penny doctors ................... 292
Periodic affections of the eye......... 228
Pharmaceutical formulæ .. ....... 515
Philosophical anatomy ............ 507
Phlebitis, Chapman on ............ 259
Phosphatic calculi, on decomp. of .. 191
Phthisis, Boudet on ... .......... 217
Phthisis, naptha in................ 267
Phthisis, statistics of .............. 464
Physical science, Smee on sources of.. 391
Physiological anatomy, Todd and Bowman's ..................... 1
Physiognomy of Serpents, Schlegel on 385
Physiology, comparative, Owen's.... 1
Physiology, animal, Carpenter's .... 1
Physiology, morality versus ........ 275
Pigeaux on diseases of the heart .... 490
Pneumonia, Watson on............ 167
Pneumonia, Addison on ........241, 464
Pneumonia, remote consequences of.. 466
Poison, definition of a ............ 432
Poisoning, statistics of ............ 435
Poisoning by Dover's powder ...... 550
Poisoning by bichromate of potass .. 551
Polypus uteri, ligature of .......... 553
Pommades .. .. ................. 515
Porrigo decalvans, origin of ........ 221
Porrigo decalvans, objections to vegetable origin of ................ 538
Potass, sulphate of, Redwood on .... 278
Potass, nitrate of, large doses ...... 549
Pouchet on fecundation............ 223
Practice of physic, Watson's........ 143
Precipitated chalk, formula for...... 551
Principles of medicine, Williams'.... 293
Profits of pharmaceutical chemists .. 280
Protein, oxydes of, in the blood .... 520
Provincial schools of pharmacy...... 280
Proximate cause of hæmorrhage .... 237
Pterygium ...................... 346
Ptisans, on the use of, in France .... 481
Puberty, acceleration of, in the female 463
Puerperal convulsions, Lever on .... 251
Puerperal convulsions, Harris on.... 268
Puerperal convulsions, Halpin on.... 270
Puerperal convulsions, Johns on .... 271
Pulmonary artery, ulceration of the.. 45
Pulsating empyema of necessity .... 540
Putrefactive emanations, influence of, on health, ...................... 417
Pyrenees, on the waters of the...... 49

Q

Quain's elements of anatomy........ 1
Quarterly table of mortality........ 541
Quina and mercury, new salt of .... 550

R

Rabies, on the cause of............ 513
Raciborski on amenorrhœa ........ 208
Raciborski on the critical age in women ........................ 211
Raciborski on the corpora lutea .... 486
Ramollissement and induration .... 145
Ranking on spermatorrhœa ........ 266
Rectum, absence of the............ 541
Redwood on sulphate of potass .... 278
Remak on menstruation .......... 484
Remuneration, med. in emigrant vess. 574
Respiratory and biliary organs, Virey on the ........................ 215
Retina, injuries of the ............ 354
Retinitis, acute .................. 350
Retinitis, sympathetic or functional.. 351
Retinitis, symptomatic ............ 351
Reveillé-Parise on health and disease 197
Rheumatism, treatment of ........ 238
Rieken on ammonia in scarlatina.... 454
Rima glottidis closed by warty vegetations........................ 538
Robinson on compression of the blood 38
Royal letter of thanks ............ 240

S.

St. George's, Dr. Jones on calculi in 39
St. George's Hospital, report from .. 251
St. Thomas's, statistics of fever in .. 531
Sanitary inquiry report.......... .. 415
Sardinia, leprosy in .............. 509
Scarlatina, Rieken on ammonia in.... 454
Schlegel on physiognomy of serpents 385
Schneiderian membrane, chronic inflammation of ................ 564
Schneiderian membrane, abscess of .. 564
Schools, naval and military ........ 355
Schools, med. in England & America, 572
Scott on cataract ................ 190
Secretion, process of.............. 25
Secretion, diseases of.............. 300
Secretion, process of .............. 508
Semeiology of the urine, Becquerel's 185
Serpents, Schlegel on physiognomy of 385
Simon on healthy and diseased bone.. 87
Simpson on infæcundity .......... 519
Simpson's new inst. for crushing calculi 576
Skull, fracture of the base of the.... 508
Sleeplessness, Graves on .......... 169
Small-pox, history of ............ 66
Smee on the sources of physical science 391
Smith's minor surgery ............ 468
Soda, carb. of, treatment of burns by 556
Softenings, cerebral .............. 459

Softenings, cerebral, colour of ...... 461
Softenings, cerebral, curability of.... 461
Solitary imprisonment with labour .. 493
Spasmodic diseases, theory of ...... 413
Spermatorrhœa, Ranking on.. ....... 266
Spermatozoa in the fluid of hydrocele 362
Spina bifida, treatment of .......... 260
Spina bifida.......................... 555
Spinal cord, gouty ramollissement of 173
Spine, lateral curvature of the ...... 221
Spirits of wine, sale of ............ 553
Statistics, medical, Foltz .......... 281
Statistics of Bethlem ............... 364
Statistics of poisoning ............ 435
Statistics of fever in St. Thomas's.... 531
Statue to Bichat.. ................. 239
Stevens on spina bifida ............ 260
Stewart on electro-puncture in hydrocele ......................... 260
Stomach of the horse, verminous tumours in the ........ ......... 236
Strangulated hernia reduced 'en masse' 45
Stricture, warm injections in ...... 261
Strumous inflammation of conjunctiva 114
Sulphate of potass, Redwood on .... 278
Suppression of urine ............... 122

T.

Tanchon on cancerous diseases...... 213
Tanning a chancre ................. 548
Tartar of the teeth, examination of the 234
Taylor on the climate of Pau ....... 49
Taylor's medical jurisprudence...... 432
Teheran, Bell on an epidemic at .... 383
Tetanic face ....................... 539
Testis, Curling on diseases of the.... 313
Testis, anatomy of the ............ 314
Testis in the fœtus......... ...... 317
Testis, descent of the..... ........ 317
Testis, congenital imperfections ..... 320
Testis, imperfect descent of the ..... 323
Thunder storms, Turley on ......... 177
Tic-douloureux of the face and head.. 236
Tice's case of bronchial calculus .... 34
Tinea capitis, Dr Wigan on ......... 265
Tinea favosa, nature of ............ 456
Todd's physiological anatomy ...... 1
Toynbee on the pathology of the ear, 369
Traill on serpents ................. 383
Transmission of hydatids by contagion 473
Trees and shrubs for cemeteries ..... 579
Treatment, hydropathic, death from.. 577
Tubercular elephantiasis, Kinnis on.. 530
Turley on thunder-storms.......... 177
Tuson on cancer .................. 547
Typhoid fever, calomel in .......... 234
Typhus fever, Lombard on ...... .. 506
Tympanitis of the uterus .......... 487
Tympanum, inflam. of membrane of, 370

U.

Ulceration of the internal jugular.... 42
Ulceration of the pulmonary artery.. 45
Ulcers of the legs, Johnson on...... 254
Umbilical cord, aneurysm of the .... 538
United States, medical education in.. 570
Urethral pains, compression for .... 514
Urinary organs, Guthrie on the .... 183
Urinary diseases, Aldridge on ...... 528
Urinary secretion, Liebig's theory of 528
Urine, suppression of.............. 122
Urine, Becquerel's semeiology of the 185
Urine, microscopic examination of the 502
Uteri, ulceration of the os and cervix 103
Uteri, os, enlargement of the glands of 104
Uteri, polypus, treated by ligature .. 553
Uterine appendages, Churchill on disdiseases of the.................. 270
Uterus, Lever on diseases of the.... 98
Uterus, inflammation of the........ 99
Uterus, corroding ulcer of the ...... 105
Uterus, carcinoma of the .......... 107
Uterus, excision of the ........... 254
Uterus, dropsy of the.............. 488
Uterus, tympanitis of the.......... 487

V.

Valerianate of zinc................. 510
Variola, chloruretted lotions in...... 513
Varus mentagra, Dauverger on...... 265
Vas deferens, deficiencies of the .... 321
Vegetable kingdom, development of.. 3
Vegetable origin of porrigo decalvans 221
Vegetable origin of porrigo decalvaas, objections to .................. 538
Velpeau on caustic in ophthalmia.... 511
Veratria in neuralgia .............. 510
Verminous tumors in stomach of horse 236
Veterinarian, the, by Youatt........ 125
Veterinary surgery, Field on........ 125
Veterinary College, charter of ...... 579
Virey on respiratory and biliary organs 215
Vis medicatrix naturæ, Cayol on the.. 219
Vocal apparatus, hysterical affection of 42

W.

Walker's occulist's vade-mecum .... 341
Walne on extirpation of ovarian cysts, 258
Warren on fissure of the palate .... 262
Warty vegetations closing rima glottidis 538
Watson's practice of physic ........ 143
Webster's case of paralysis ........ 32
Webster on the statistics of Bethlem 364
Webster on insanity .............. 365
Wigan on tinea capitis ........... 265
Williams on the pathology of cells .. 246
Williams on extirpation of the superior maxilla.................. 249
Williams's principles of medicine.... 293

Y.

Yellow bark....................... 277
Youatt's veterinarian .............. 125

Z.

Zinc, valerianate of............... 510
Zinc, chloride of, in cancer ........ 547

### BIBLIOGRAPHICAL RECORD.

1. The Popular Cyclopædia of Natural Science. Parts I. and II., Animal Physiology. By W. B. CARPENTER, M. D.—W. S. Orr and Co. October, 1843.
☞ *A most valuable composition.*

---

2. Pharmaceutical Journal and Transactions. Edited by JACOB BELL. October, November, December, 1843.

---

3. Posthumous Extracts from the Veterinary Records of the late JOHN FIELD. Edited by his Brother, WILLIAM FIELD, Veterinary Surgeon, London. Octavo, pp. 236. Longman and Co. Nov. 1843.

---

4. Cataract, and its Treatment; comprising an easy Mode of dividing the Cornea for its Extraction, and appropriate Means for removing the different Forms of that Affection, &c. By JOHN SCOTT, Senior Surgeon to the London Ophthalmic Hospital, &c. Octavo, pp. 69. Churchill, 1843.

---

5. The British Journal of Homœopathy. Vol. I. 1843. Edited by J. J. DRYSDALE, M.D., J. R. RUSSELL, M.D., and FRANCIS BLACK, M.D. Octavo, pp. 422. Leath, St. Paul's Churchyard. Bailliere, Regent Street.

6. The New York Journal of Medicine and the Collateral Sciences. Edited by SAMUEL FORRY, M.D. Published bimonthly. Vol. I. No. 1, July 1843.
☞ *In exchange.*

---

7. Medico-Chirurgical Transactions, published by the Medical and Chirurgical Society of London. Second Series, Vol. VIII. 1843.

---

8. A Practical Treatise on Organic Diseases of the Uterus; being the Prize Essay to which the Medical Society of London awarded the Fothergillian Gold Medal for 1843. By J. C. W. LEVER, M.D. Assist.-Accoucheur at Guy's Hospital, &c. Octavo, pp. 240. Longman and Co. 1843.

---

9. Clinical Remarks on certain Diseases of the Eye, and on Miscellaneous Subjects, Medical and Surgical, including Gout, Rheumatism, Fistula, Cancer, Hernia, Indigestion, &c. By JOHN CHARLES HALL, M.D. M.R.C.S., &c. Octavo, pp. 228. Churchill, London, 1843.

---

10. On the Nature and Treatment of Stomach and Renal Diseases; being an Inquiry into the Connexion of Diabetes, Calculus, and other Affections of the Kidney and Bladder with Indigestion. By William Prout, M.D. F.R.S., &c. Fourth Edition, revised. Churchill, London. 1843.

11. Elementary Instruction in Chemical Analysis. By Dr. C. Remigius Fresenius, Chemical Assistant in the Laboratory of the University of Giessen. With a Preface by Professor Liebig. Edited by J. Lloyd Bullock, Member of the Chemical Society, &c. Octavo, pp. 284. Oct. 1843. Churchill, London.

12. The Principles of Medicine: comprising General Pathology and Therapeutics; and a brief general View of Etiology, Nosology, Semeiology, Diagnosis, and Prognosis. By Charles J. B. Williams, M.D., F.R.S., Professor, &c. in University College, and First Physician of the Hospital, &c. 8vo, pp. 390. Churchill, London, 1843.

13. The Endemic Influence of Evil Government, illustrated in a View of the Climate, Topography, and Diseases of the Island of Minorca, &c. By J. M. Foltz, M.D. Surgeon in the American Navy. Octavo, New York, 1843.

14. The Anatomy of the Arteries. By Richard Quain, Esq., Professor of Anatomy, University College, &c. Part XVI. Five Plates, with Letter-press, price 12s. 1843.

15. An Essay on Thunder-storms, their Phenomena, and Means of Safety from their Effects. By E. A. Turley, M.D., &c. Worcester, Nov. 1843. Pp. 35.

16. Remarks on the School of Instruction for Military and Naval Surgeons. In a letter to the Right Hon. Sir Robert Peel. By Sir George Ballingall, M.D., Regius Professor, &c. 1843.

17. A Practical Manual of the General, Chemical, and Microscopic Characters of the Blood, and of the Secretions of the Human Body, &c. By John William Griffith, M.D. Small Octavo, pp. 64. R. & J. Taylor. 1843.

18. The Oculist's Vade Mecum; a complete Practical System of Ophthalmic Surgery. With numerous Wood-cuts. By John Walker, Surgeon to the Manchester Eye Hospital, &c. Longman & Co. 1843.

19. Memoire sur deux Instruments nouveaux destinas a l'Extraction et l'Abaissement de la Cataract. Par le Docteur Alphonse, de Grand Boulogn. Marseille, 1843.

20. An illustrated Catalogue of the Works published by John Van Voorst, Paternoster Row, London. 1843.

21. The New York bi-monthly Journal of Medicine and the Collateral Sciences. Edited by S. Forry, M.D. Sept. 1843.

☞ *In exchange.*

22. Introduction to the Study and Practice of Midwifery. By William Campbell, and by Alex. D. Campbell, B. A., Trinity College, Dublin, &c. Second Edition, pp. 804. Edinb. Whyle, 1843.

☞ *Much improved.*

23. Researches on the Decomposition and Disintegration of Phosphatic Vesical Calculi, and on the Introduction of Chemical Decomponents into the Living Bladder. By S. Elliott Hoskins, M.D. (From the Philosophical Transactions.)

24. Caloric, its Mechanical, Chemical, and Vital Agency in the Phenomena of Nature. By Samuel L. Metcalfe, M.D. Two Vols. 8vo. Pickering, London, 1843.

25. The Surgeon's Vade Mecum. By Robert Druitt, Surgeon. Octavo, pp. 572. Renshaw, London, 1843.

26. Principles of Forensic Medicine. By William A. Guy, M.B. Part II. London, H. Renshaw. 1843.

27. The Cold Water Cure, as practised by Vincent Priessnitz, at Graafenberg, in Silesia, with an Account of Cases. By Richard Beamish, Esq. F.R.S., &c. 8vo, pp. 104 Highley, 1843.

28. The Physiology of Inflammation, and the Healing Process. By Benjamin Travers, F.R.S., &c. Octavo, pp. 226. 1843.

29. A concise Exposition of Homœopathy—its Principles and Practice, &c. By George Newman, M.R.C.S. 8vo, 1843. Leath, Publisher.

30. The Principles of Physiology applied to the Preservation of Health, &c. By Andrew Coombe, M.D. With Fifteen Woodcuts. Twelfth Edition, enlarged.

Maclachlan, Stewart, and Co. Ed. 1843. Price 2s. 6d.

---

31. On Superstitions connected with the History and Practice of Medicine and Surgery. By T. J. PETTIGREW, F.R.S. &c. Octavo, pp. 167. Churchill, London. Nov. 1843.

---

32. Gonorrhœa, and its Consequences; with a short Historical Sketch of the Venereal Disease. By G. B. CHILDS, M R.C.S. Highley, London. 1843.

---

33. The Hand-book of Hydropathy, Professional and Domestic. By Dr. J. WEISS. Octavo. Madden and Co. 1843.

---

34. The Pharmaceutical Journal and Transactions, &c. Dec. 1. 1843.

---

35. A Dictionary of Practical Medicine, &c. By JAMES COPLAND, M.D. Part IX. London, Longman and Co. Dec. 1843.

---

36. Removal of a Dropsical Ovarium. By GEO. SOUTHAM, Surgeon to the Salford Royal Dispensary. From the Medical Gazette. Dec. 1843.

---

37. Elements of Natural Philosophy; being an Experimental Introduction to the Study of the Physical Sciences. By Dr. GOLDING BIRD, A.M., M.D. Second Edit. revised and enlarged. Churchill, Dec. 1843.

---

38. The Medical Almanack or Calendar of Medical Information, for the Year 1844. Being Bissextile, or Leap Year. Churchill, 1843. Price One Shilling.

☞ *A most valuable compilation.*

---

39. Observations on Pneumonia and its Consequences. With Plates. By THOMAS ADDISON, M.D. Dec. 1843.

---

40. The Sources of Physical Science. Being an Introduction to the Study of Physiology through Physics, &c. By ALFRED SMEE, F.R.S., &c. Octavo, pp. 296. Renshaw, Strand. Dec. 1843.

---

41. Cases of Puerperal Convulsions, with Remarks. By Dr. Lever. [From Guy's Hospital Reports.]

---

42. Guy's Hospital Reports. (Second Series.) No. II. October, 1843. Edited by Drs. BARLOW, COCK, BIRKETT, BROWNE, and POLAND. Price Nine Shillings. Highley, 32, Fleet Street. 1843.

---

43. A Manual of Medical Jurisprudence. By ALFRED S. TAYLOR, Lecturer on Medical Jurisprudence, &c. in Guy's Hospital. Octavo, pp. 677. Churchill, London. Dec. 1843.

☞ *This valuable work will be noticed in our next number.*

---

44. Further Contributions to a Knowledge of the Influence of Employments upon Health. By WILLIAM AUGUSTUS GUY, M.B., Cantab. Professor of Forensic Medicine, King's College, &c. From the Quarterly Journal of the Statistical Society.

---

45. Observations on the Proximate Cause of Insanity. By JAS. SHEPPARD, M.R.C.S. Being an attempt to prove that Insanity is dependent on a Morbid Condition of the Blood. Small 8vo, pp. 104. Longman and Co. 1843.

---

46. The Influence of Climate, and other Agents, on the Human Constitution, with reference to the Causes and Prevention of Disease among Seamen: with Observations on Fever in general, and an Account of the Epidemic Fever of Jamaica. By ROBERT ARMSTRONG, M.D., F.L.S., Deputy Inspector of Hospitals, &c. Octavo, pp. 207. Longman and Co. Dec. 1843.

---

47. The Anatomy, Physiology, and Pathology of the Human Teeth; with the most approved Methods of Treatment; including Operations and the Method of making and setting Artificial Teeth. With 30 Plates. By PAUL P. GODDARD, M.D., M.A.N.S., &c. Demonstrator of Anatomy in the University of Pennsylvania, &c. Aided in the Practical Part by E. PARKER, Dentist. Quarto. Philadelphia, Caray and Co. 1844.

---

48. Elements of Physiology, for the Use of Students, and with particular Reference to the Wants of Practitioners. By RUDOLPH WAGNER, M.D., Professor of Comparative Anatomy and Physiology in the University of Gottingen, &c. Part III. (completing the Special Physiology), on Sensation and Motion. From the German, with Additions. By ROBERT WILLIS, M.D., Member of the Royal College of Physicians, &c. With illustrative Wood-engravings. London, Sherwood, Gilbert, and Piper, Paternoster Row. 1844.

